# Association between Nonalcoholic Fatty Liver Disease and Endocrinopathies: Clinical Implications

**DOI:** 10.1155/2021/6678142

**Published:** 2021-01-11

**Authors:** Ana-Maria Singeap, Carol Stanciu, Laura Huiban, Cristina Maria Muzica, Tudor Cuciureanu, Irina Girleanu, Stefan Chiriac, Sebastian Zenovia, Robert Nastasa, Catalin Sfarti, Camelia Cojocariu, Anca Trifan

**Affiliations:** ^1^Department of Gastroenterology, “Grigore T. Popa” University of Medicine and Pharmacy, Iasi 700115, Romania; ^2^Institute of Gastroenterology and Hepatology, “St. Spiridon” Emergency Hospital, Iasi 700111, Romania

## Abstract

Nonalcoholic fatty liver disease (NAFLD) has a rising prevalence worldwide. Its potential for evolution towards liver cirrhosis and hepatocellular carcinoma, as well as associations with extrahepatic manifestations, represents a double burden for patients and physicians alike. Recently, there has been increasing evidence of the association between NAFLD and a number of endocrinopathies, such as hypothyroidism, polycystic ovarian syndrome (PCOS), hypopituitarism, growth hormone deficiency (GHD), hypogonadism, and hypercortisolism. Definite correlations are supported by clear evidence so far, but further studies are needed in order to completely clarify the pathogenic mechanisms and, especially, to identify therapeutic implications. In this review, we present the main relationships between NAFLD and endocrinopathies, emphasizing the reciprocal causality, evolutive interconnections, and current clinical scenarios of presentations of which the clinicians should be aware.

## 1. Introduction

Nonalcoholic fatty liver disease (NAFLD) is one of the most important nonneoplastic pathologies in contemporary medicine, being the most common cause of chronic liver disease worldwide. It is characterized by the accumulation of fat in the liver, histologically being identical to alcoholic liver disease, in patients without significant alcohol consumption [1].

The clinical importance of NAFLD is related to its prevalence of up to 30% in the general population, thus exceeding that of viral hepatitis and alcoholic liver disease [2, 3]. It is worrying that the disease includes not only a form considered benign (hepatic steatosis) but also progressive forms (steatohepatitis with or without fibrosis) possibly evolving towards liver cirrhosis and, in some cases, hepatocellular carcinoma [4]. Moreover, the pathogenic mechanisms of progression from the simple form to the aggressive ones are not completely elucidated due to their complexity, through the involvement of multiple processes as well as metabolic, immunological, and genetic imbalances [5]. NAFLD is a topical issue in terms of diagnosis and treatment for a variety of specialties. One element that certifies the importance of nonalcoholic fatty liver is the fact that, in the coming years, this pathology is expected to become the first indication for liver transplantation, surpassing viral liver cirrhosis of any etiology [6].

In recent years, there has been increasing evidence of the association between NAFLD and a number of endocrinopathies, such as hypothyroidism, polycystic ovarian syndrome (PCOS), hypopituitarism, growth hormone deficiency (GHD), hypogonadism, and hypercortisolism [7]. The relationship between NAFLD and these hormonal abnormalities is not yet completely understood as their role in the pathogenesis of NAFLD has not been established so far.

However, despite the fact that NAFLD is an increasingly common disease, it is often overlooked by endocrinologists and is assessed only by hepatologists. Given NAFLD's long-term clinical impact, it is important for practicing physicians, endocrinologists, and hepatologists to detect forms of NAFLD associated with endocrine diseases.

In this review, we tried to include the most important up-to-date information on the association of nonalcoholic fatty liver disease and various endocrinopathies, with specific reference to their epidemiology, pathophysiological mechanisms, and treatment principles.

## 2. NAFLD and Hypothyroidism

The thyroid gland plays a key role in the regulation of various metabolic processes and its dysfunction is linked to diverse diseases. Disorders in thyroid hormones concentration may lead to insulin resistance, obesity, and hyperlipidemia, which are well-known risk factors for the development of NAFLD [8]. Thyroid-stimulating hormone (TSH) can directly increase hepatic gluconeogenesis and decrease 3-hydroxy-3-methylglutaryl coenzyme A (HMG-CoA) reductase phosphorylation inducing hypercholesterolemia [9]. Moreover, oxidative stress is a common pathway for both hypothyroidism and NAFLD [10]. In 2014, in a systematic review, the authors found a prevalence of hypothyroidism ranging between 15.2% and 36.3% among patients with NAFLD [11], while the national estimated prevalence of hypothyroidism in the United States population was 3.7% [12]. In one particular study which analysed patients with biopsy-proven NAFLD, the prevalence of hypothyroidism was higher compared to matched controls (21% vs. 9.5%, resp.), regardless of age, obesity, hyperlipidemia, and diabetes [13]. This association seems to be more than a mere coincidence, since hypothyroidism may be involved in the development of NAFLD. Thus, a recent systematic review and meta-analysis including over 42000 patients from 13 studies found a high correlation between hypothyroidism and NAFLD, providing strong epidemiological evidence regarding the risk for NAFLD for both subclinical and overt hypothyroidism, compared to euthyroid subjects [14]. Overt hypothyroidism, defined as increased TSH and low free T4 (FT4), was more significantly correlated with NAFLD than subclinical hypothyroidism, stated as increased TSH and normal FT4, probably due to the combined concomitant effects of low thyroid hormones and higher TSH level [14]. Subclinical hypothyroidism was connected to NAFLD in a dose-dependent manner; even in the range of normal TSH levels, association with NAFLD was reported independently of other recognized metabolic risk factors [15]. Moreover, “low-normal” thyroid function was reported as a risk factor for advanced fibrosis [16]. Recently, the role of thyroid hormone receptor in hepatic stellate cells activation was postulated [17], but it still remains unclear if or how exactly thyroid dysfunction is supposed to accelerate the progression of NAFLD to steatohepatitis and, consequently, to advanced fibrosis. Thyroid hormone replacement therapy leads to a significant decrease of serum lipids and has a favourable effect on overweight or obesity [18]. Levothyroxine administered for 15 months in patients with subclinical hypothyroidism showed benefit on serum transaminases and ultrasound-diagnosed NAFLD [19]. Decrease of hepatic fat content measured by magnetic resonance spectroscopy was demonstrated after low-dose levothyroxine administered for 4 months in patients with normal thyroid function, type 2 diabetes, and NAFLD [20]. However, it is not yet known if thyroid replacement therapy in patients with NAFLD will improve the current status of the disease or stop its progression, as more studies are needed to elucidate the interrelation between NAFLD and hypothyroidism. Nevertheless, until further evidence, NAFLD patients' surveillance should be carried out by annual TSH testing.

## 3. NAFLD and Polycystic Ovarian Syndrome

Polycystic ovarian syndrome (PCOS) is a reproductive disease characterized by hyperandrogenism with polycystic ovarian and oligomenorrhea, after exclusion of other endocrine disorders [1]. PCOS is the most common cause of anovulatory infertility, affecting 5% to 18% of the reproductive aged women worldwide, while it may also lead to additional health problems in adulthood, with long-term consequences [21]. This disease is associated with risk factors for cardiovascular disease, obesity (60%), hepatic steatosis (50%), and insulin resistance (IR) (70%) [22].

An increasing number of cohort studies strongly suggest that the prevalence of NAFLD is remarkably high in young women with PCOS, regardless of the presence of obesity or other features of the metabolic syndrome [4]. Brown et al. described NAFLD for the first time after a liver biopsy for a 24-year-old woman with PCOS, obese, without a history of alcohol consumption, diabetes, or other known liver diseases which are consistently associated with increased transaminases [23].

Lim and Bernstein showed that the prevalence of NAFLD in PCOS varies from 35 to 70%, compared with 20 to 30% in women that do not present with PCOS, of similar age, BMI, and hip circumference [24]. Ramezani-Binabaj et al. reported in a meta-analysis that patients with PCOS had a 3.93-fold increased risk of coexisting NAFLD independent of BMI [25]. In particular, some case-control studies have also reported that PCOS is very common among young women with biopsy-proven NAFLD. In these patients, the prevalence of PCOS ranged from about 50% to 70%, at the same time being more likely to have the most severe histological forms of NAFLD [26]. A recent meta-analysis of 17 observational studies, which included 2734 women with PCOS and 2561 healthy women of similar age and BMI, showed that young women with PCOS had twice the risk of prevalent NAFLD than control women [27].

According to the Rotterdam diagnostic criteria for the three PCOS phenotypes (classical, ovulatory, and normal androgen), studies have shown that the risk of insulin resistance and metabolic syndrome is the highest in women with PCOS and classical phenotype, intermediate in those with ovulatory phenotype, and the lowest in those with normo- and rogenic phenotype [28, 29].

Over time, there has been an ongoing debate about mechanisms underlying the alleged involvement of PCOS in NAFLD development and progression. There is ample scientific evidence to suggest that PCOS and NAFLD have common multifactorial pathophysiological mechanisms, representing a complex interaction between abdominal adiposity/overweight/obesity, systemic insulin resistance, chronic inflammation, and hyperandrogenism [4]. In 1980, Burghen et al. showed that patients with obese PCOS associate hyperinsulinemia, a hypothesis later confirmed in other studies, which have even proved that it is independent of obesity [30, 31]. The association of PCOS with increased tolerance to glucose and diabetes, abdominal adiposity, and dyslipidaemia, as well as with metabolic syndrome, led to its classification as a metabolic disorder [32].

The main presumed reasons why PCOS women may have low insulin sensitivity include both defects in the insulin receptors found on the surface of the ovaries and irregular insulin signalling which increases androgen production in theca cells, the primary source of excessive androgen biosynthesis in women with PCOS [33]. Thus, IR leads to compensatory hyperinsulinemia, which stimulates theca cells in LH-sensitized ovaries to secrete testosterone and androstenedione. Baranova et al. showed in a systematic analysis that IR is present in 50%–80% of women with PCOS and NAFLD [34]. In addition, multiple studies have shown that PCOS women with hepatic steatosis have high levels of IR compared to PCOS women without steatosis [1, 3].

Another theory that explains the pathogenic mechanism between NAFLD and women with PCOS refers to the effects of hyperandrogenism on low-density lipoprotein (LDLR) receptors, in which androgens suppress LDLR gene transcription to prolong the half-life of very low-density lipoproteins (VLDL) and LDL, thus causing the accumulation of lipids in the liver [35]. Kumarendran et al. demonstrated in a retrospective cohort study, which included over 63000 women with PCOS and 121000 age-, BMI-, and location-matched women, that PCOS subjects had an increased incidence of NAFLD, hyperandrogenism being a risk factor for NAFLD development in women with PCOS [36].

Currently, more studies are needed to demonstrate the best management of NAFLD in women with PCOS. The first recommended therapeutic option for NAFLD in women with PCOS is to change their lifestyle based on a low-calorie diet and increased physical activity. The second option is the association between a lifestyle change and metformin [29, 37]. Evidence has recently emerged of the benefits of pioglitazone, liraglutide, and other glucagon-1 peptide-1 receptor agonists for decreasing intrahepatic fat content in the treatment of women with PCOS and NAFLD [38, 39].

Given the increasing prevalence of NAFLD in young women with PCOS and the high risk of developing long-term liver complications, systematic screening of NAFLD in patients with PCOS should be considered, especially whenever hyperandrogenism and metabolic syndrome are present. Unfortunately, the optimal method of screening in this population is currently unknown, but monitoring of aminotransferase and hepatic steatosis levels by ultrasound, especially in those with metabolic syndrome, can be a start.

## 4. NAFLD and Hypopituitarism

Hypopituitarism is a chronic endocrine disorder defined as the lack or diminution of pituitary gland function, due to pituitary or hypothalamic pathologies. There are two types of hypopituitarism, classified by the main causal factor: primary hypopituitarism explained by an intrinsic illness of the pituitary gland (neoplasia, ischemia, infectious of infiltrative diseases, genetic syndromes, and immunological or inflammatory pathologies) and secondary hypopituitarism due to pituitary stalk, hypothalamus, or other central nervous systems (tumoral, infiltrative, traumatic, infectious, and nutritional) disorders [40]. The anterior pituitary part, known as adenohypophysis, secretes into the systemic circulation of the following hormones: thyroid-stimulating hormone (TSH), gonadotropins, somatotropin or growth hormone (GH), corticotropin or adrenocorticotropic hormone (ACTH), and prolactin.

Increased prevalence of metabolic syndrome and NAFLD was described in hypopituitarism, with cardiovascular disease notably higher, and significant premature mortality due to cardiovascular disease [41, 42]. From the classical point of view, this association between hypopituitarism, metabolic syndrome, and NAFLD is linked to lipid disequilibrium and liver fat accumulation. In hypopituitarism, there is a decreased high-density lipoprotein cholesterol level and an increased low-density/high-density lipoprotein ratio [42]. Thus, metabolic syndrome in patients with hypopituitarism is specifically distinguished by lower high-density lipoprotein level [43]. More recent arguments implicate leptin, an anorectic hormone, and the leptin resistance in hypopituitarism, in the pathogenesis of NAFLD, via insulin resistance, hyperphagia, and obesity [44, 45]. In an observational study including 69 patients with hypopituitarism without hormonal replacement therapy, the prevalence of ultrasound-diagnosed NAFLD was 77% compared to 12% in controls [46]. One cohort cross-sectional study showed that nontreated female patients with hypopituitarism have a double risk for cardiovascular mortality compared to the general population [42]. Hypopituitarism may be a rare cause of rapidly progressive NAFLD, as shown by a study reporting cases of young patients with fast deterioration towards cirrhosis in conjunction with hypopituitarism [47]. Another recent retrospective study including surgical and nonsurgical hypopituitarism patients revealed that liver fibrosis grade had a rapid increase in surgery cases of NAFLD patients with hypopituitarism, which correlated significantly with leptin serum levels [48]. Further studies are needed in order to completely describe the NAFLD and hypopituitarism relation and enforce timely therapeutic measures.

## 5. NAFLD and Growth Hormone Deficiency

Growth hormone (GH) is under the control of the hypothalamic-pituitary somatotropic axis, with an important metabolic role in the liver, adipose tissue, and skeletal muscle by regulating glucose and lipid metabolism, body composition, and growth in both children and adults [49]. Therefore, GH acts in different tissues through complex mechanisms, either directly by interaction with the GH receptor or indirectly through its mediator insulin-like growth factor-1 (IGF-1) [50].

Growth hormone deficiency (GHD) is a rare disorder characterized by the inadequate secretion of GH from the anterior pituitary gland [1]. GHD can be congenital, due to genetic mutations or structural defects of the brain, acquired, secondary to a tumor, trauma, infection or radiation therapy, or idiopathic. GHD with childhood onset is manifested by growth retardation and short stature inconsistent with child's chronological age. GHD with adult onset is most commonly secondary to brain trauma or a pituitary tumor but can also be idiopathic. It is manifested by reduced muscular endurance, lipid abnormalities, insulin resistance, and impaired cardiac function [1].

The role of GH in the liver is known to stimulate gluconeogenesis and glycogenolysis, contrasting insulin signalling. Kim and Park demonstrated, in a recent study, that GHD causes insulin resistance, probably due to an increased flow of free fatty acids and the inhibition of glycogen synthesis, but the exact mechanisms remain unknown [51].

Recent studies have shown that adult patients with untreated GHD have a phenotype similar to that of the metabolic syndrome, which is strongly associated with NAFLD [49, 51], suggesting a possible association between GHD and NAFLD [52]. Based on this hypothesis, numerous studies have consistently shown that patients with NAFLD associate lower levels of serum GH compared to controls without NAFLD [53, 54]. At the same time, a correlation was noticed between lower GH levels and histological severity of NAFLD [54, 55]. A worrying fact is that GHD is a risk factor in the development and progression of secondary forms of NAFLD, which are not reversible on account of lifestyle changes [56, 57].

In a retrospective study, Adams et al. demonstrated a prevalence of NAFLD of approximately 2% in patients with hypothalamic or pituitary disorders [58]. More specifically, in patients with GHD, a 6.4-fold increased prevalence of NAFLD was found compared to age, sex, and appropriate BMI controls [46]. Ichikawa et al. demonstrated that patients with GHD had a significantly increased risk of NAFLD compared with those with pituitary dysfunction without GHD [59]. Also, Hong et al. in a case-control study showed that serum GH levels were lower in patients with NAFLD and the prevalence of NAFLD was significantly higher in men with hypopituitarism than in healthy controls [60].

Given the metabolic effects of GH on adipose tissue and liver, the main pathophysiological mechanism linking GHD to NAFLD is insulin resistance and increased lipogenesis, common pathways found in the development of NAFLD [1]. In addition, NAFLD development and progression in patients with GHD is also explained by the increased level of proinflammatory cytokines in the context of the systemic inflammatory status [1].

Therefore, based on the reported data, screening for NAFLD is recommended in all patients with GHD. Although study results are discrepant, given the beneficial effects on insulin resistance, inflammation, and fibrosis, the administration of exogenous GH for the treatment of secondary GHD NAFLD is justified [57, 61].

## 6. NAFLD and Hypogonadism

Hypogonadism is a congenital or acquired condition characterized by decreased reproductive function in both men and women, regardless of the cause [1]. Hypogonadism can be primary or secondary. Primary or hypergonadotropic hypogonadism (peripheral, gonadal) consists in an inadequate gonads response to gonadotropins resulting in decreased sex hormone levels and increased gonadotropin levels (luteinizing hormone, LH, and follicle-stimulating hormone (FSH)). Secondary or hypogonadotropic (central) hypogonadism refers to the inability of the hypothalamus or pituitary gland to produce enough FSH and LH and is characterized by low concentrations of sex hormones, FSH, and LH [62].

The main causes of primary hypogonadism are autoimmune diseases, genetic disorders, infections, liver and kidney disease, radiation, surgery, and trauma. Causes of central hypogonadism include pituitary hemorrhage, anorexia nervosa, radiation, medications (glucocorticoids, opiates), iron excess, surgery, trauma, and tumors [62].

Recently, numerous studies have reported a strong bidirectional association between NAFLD and hypogonadism in both genders [63–65]. Seo et al. demonstrated in a cross-sectional study on 1944 men without alcohol consumption that serum testosterone levels in NAFLD patients were reduced compared to non-NAFLD. At the same time, the higher the risk of NAFLD, the lower the testosterone level [66]. Moreover, Gild et al. demonstrated in a cohort of 380669 patients an improvement in NAFLD status in hypogonadic men who received hormone replacement therapy [64]. They also showed that there is a higher risk of NAFLD in men with prostate cancer and hypogonadism induced by androgen deprivation therapy [64]. Men with hypogonadism have higher noninvasive indices of NAFLD (hepatic steatosis index and triglyceride-to-HDL-C ratio), compared to men with high testosterone levels [67].

Also, in women with hypogonadism, an increased prevalence of serum liver enzymes has been demonstrated, surrogating indices of NAFLD [68, 69]. Also, it is known that estrogen deficiency, which can occur in hypogonadism or in postmenopausal women, is associated with a higher prevalence of NAFLD and advanced liver fibrosis. Prolonged estrogen deficiency has been associated with a long-term risk of advanced liver fibrosis [65].

The main pathophysiological mechanisms linking hypogonadism to NAFLD are complex and still under investigation. The main factors involved are estrogen deficiency [70], intestinal dysbiosis which causes a decrease in androgen hormones [71], and low levels of dehydroepiandrosterone, a hormone that affects insulin resistance and fibrogenesis [72] and adiposity, which is dependent on sex hormones and increases the risk of NAFLD development and progression through decreasing glucose tolerance [73].

General therapeutic measures are based on lifestyle changes, through dietary restrictions and regular exercise. Specific treatment measures depend on gender, so that in male hypogonadism, the treatment of choice is testosterone administration, while in women, hypogonadism is treated with estrogen replacement therapy [1].

In conclusion, it is recommended that patients with hypogonadism and NAFLD be monitored by both endocrinologist and hepatologist in order to prevent both the progression of liver damage as well as extrahepatic complications of NAFLD [74].

## 7. NAFLD and Hypercortisolism

Glucocorticoids (GCs) are produced by the adrenal gland under the control of pituitary ACTH secretion. High levels of circulating glucocorticoids favour liver gluconeogenesis and lower insulin sensitivity and are correlated with visceral obesity, dyslipidaemia, diabetes, arterial hypertension, coronary artery disease, and NAFLD [75]. Because of the similar metabolic disturbances and clinical features, there is an apparent overlap between metabolic syndrome and Cushing syndrome. However, the pathogenic pathway of GCs towards metabolic syndrome is still incompletely established. Excess tissue GCs are incriminated in the genesis of a metabolic syndrome rather than increased plasmatic GCs levels, based on the fact that circulating plasma corticosterone levels as well as urinary corticosterone levels is usually normal in patients with obesity and type 2 diabetes [76]. Active corticosterone (cortisol) becomes available from inert cortisone via specific prereceptor enzyme 11*β*-hydroxysteroid dehydrogenase-1 in key metabolic target tissues such as visceral adipose tissue and liver; the components of the metabolic syndrome may be a consequence of the increase in locally available glucocorticoids and not of the high circulating corticoid levels [77]. In truth, only 20% of patients with Cushing's syndrome have liver steatosis, measured by liver/spleen attenuation on CT scanning [78]. Conversely, even if it has been proved that NAFLD patients have hormonal abnormalities such as higher urinary free cortisol concentrations and lower dexamethasone suppression of plasma cortisol, there is no evidence to sustain an associated Cushing-like syndrome [79].

GCs' metabolism has an adaptive power since there are differences between uncomplicated steatosis and steatohepatitis; in simple steatosis, there is an increased clearance of cortisol as a protective mechanism trying to restrict lipid accumulation while, in steatohepatitis, the 11*β*-HSD1 has an increased activity in order to limit hepatic inflammation [80].

Base on the strength of its promoting role in lipogenesis, deletion or pharmacological inhibition of 11*β*-HSD1 could decrease hepatic steatosis [81], as favourable results on lipid profile and glycemic control are documented in patients with diabetes [82]. Even though there are clear associations between hypothalamic-pituitary-adrenal (HPA) axis disorders and NAFLD, further clarifications are needed especially in terms of targeted therapeutical directions.

## 8. Conclusions

The expanding prevalence of NAFLD and its relationships with many endocrine diseases are real challenges for clinicians nowadays. NAFLD has a potential for evolving towards severe complications such as cirrhosis or hepatocellular carcinoma but may also expose to various extrahepatic manifestations, adding to the burden weighing on patients and practicing physicians alike. Irrespective of the fact that NAFLD patients are at risk of developing specific endocrine pathologies or, contrarily, some endocrinopathies predispose patients at risk of developing NAFLD, awareness about the interconnections between NAFLD and endocrinopathies is of utmost importance, in order to provide to all patients appropriate surveillance and optimal therapeutical approach.

## Data Availability

The data used to support this study are included within this article.
